# Oral Administration of Probiotics Reduces Chemotherapy-Induced Diarrhea and Oral Mucositis: A Systematic Review and Meta-Analysis

**DOI:** 10.3389/fnut.2022.823288

**Published:** 2022-02-28

**Authors:** Jing Feng, Min Gao, Chengcheng Zhao, Jian Yang, Haiyan Gao, Xin Lu, Rong Ju, Xiuwei Zhang, Yunlei Zhang

**Affiliations:** ^1^Department of Respiratory and Critical Care Medicine, The Affiliated Jiangning Hospital of Nanjing Medical University, Nanjing, China; ^2^Department of Biomedical Engineering, School of Biomedical Engineering and Informatics, Nanjing Medical University, Nanjing, China; ^3^Central Laboratory, Translational Medicine Research Center, The Affiliated Jiangning Hospital of Nanjing Medical University, Nanjing, China; ^4^Department of Obstetrics and Gynecology, The Affiliated Jiangning Hospital of Nanjing Medical University, Nanjing, China

**Keywords:** probiotics, cancer, chemotherapy, diarrhea, oral mucositis

## Abstract

**Background:**

Chemotherapy generally causes serious diarrhea and oral mucositis in cancer patients, and subsequently affects treatment. Oral administration of probiotics provides a therapeutic choice to address these limitations. This study aims to conduct a systematic review and meta-analysis on the efficacy of oral probiotic use in the management of the chemotherapy-induced adverse reactions, and to summarize the mechanisms underlying the action.

**Methods:**

We searched PubMed, Embase, ClinicalTrials.gov, and Web of Science from the start of the study to its completion on Dec. 31, 2021. Risk of bias was assessed using Cochrane Collaboration's Tool. Statistical analysis of the acquired data was performed via the RevMan and the Stata Statistical Software. The protocol was registered in the International Prospective Register of Systematic Reviews (PROSPERO registration number: CRD42020220650).

**Results:**

Twelve randomized controlled trials including 1,013 patients were recruited and analyzed via the standard procedure of meta-analysis. In contrast to the control group, orally taking probiotics significantly decreased the risk of chemotherapy-induced diarrhea (≥ 1 grade) (*RR* = 0.70; 95% Cl: 0.56, 0.88; *P* = 0.002) and oral mucositis (≥ 1 grade) (RR: 0.84; 95% Cl: 0.78, 0.91; *P* < 0.00001) at all grades. Further analysis found that severe diarrhea (≥ 2 grades) (RR: 0.50; 95% Cl: 0.32, 0.78; *P* = 0.002) and severe oral mucositis also significantly declined (≥ 3 grades) (RR: 0.66; 95% Cl: 0.55, 0.79; *P* < 0.00001) after oral probiotic use. Interestingly, the beneficial effects of probiotics displayed statistically significant only in Asian patients. Importantly, the more species of bacteria they took, the lower the incidences of the adverse reactions occurred. We used Egger's test *P* value to confirm that there is no publication bias.

**Conclusions:**

This meta-analysis demonstrated that orally administrated probiotics has a potential to decrease chemotherapy-induced diarrhea and oral mucositis incidences. However, the efficacy of oral probiotic use against the adverse reactions needs to be further verified through more clinical trials, and the species and number of probiotics have to be optimized and standardized prior to clinical applications.

**Systematic Review Registration:**

https://www.crd.york.ac.uk, identifier: 220650.

## Introduction

Diarrhea and oral mucositis elicited by chemotherapy lead to interruptions or changes to the therapeutic regime and subsequently affect patient prognosis and overall survival. Current clinical practice guidelines for treating chemotherapy-induced diarrhea mainly rely on diet modification and heteropathy treatment using drugs (including loperamide, octreotide, and opium tinctures) (1). However, the treatments are often accompanied by additional gastrointestinal symptoms, including stomach pain, diarrhea, and vomiting (2, 3). Also, chemotherapy-induced oral mucositis does not have any evidence-based clinical management regimes, although some clinical strategies have been recommended by the Multinational Association of Supportive Care in Cancer and International Society of Oral Oncology (MASCC/ISOO) (4). Therefore, it is crucial to develop novel drugs or treatment strategies to decrease diarrhea and oral mucositis as a result of chemotherapy.

Increasing evidences demonstrate the key role of probiotics in the management of patients with inflammatory bowel disease, allergy, autoimmune disease, and cancer (5), presenting a new avenue to address these limitations. The bacteria can stimulate the proliferation of beneficial bacteria of intestinal microbiota, thus maintaining a healthy intestinal environment or improving diseased one (6, 7). Actually, daily probiotic use succeeded in reducing the adverse reactions of chemotherapy in several clinical trials (8–10). For example, cervical and colorectal cancer patients who took probiotic capsules exhibited a lower incidence of diarrhea during chemotherapy than those who used the placebos (11, 12). Orally administrated probiotics obviously decreased grade III and IV oral mucositis in chemotherapy-treated patients with head and neck squamous cell carcinoma and nasopharyngeal carcinoma (13). In addition, postoperative complications due to infection were significantly reduced in cancer patients because of oral probiotic use (14). The mechanisms underlying the beneficial effects mainly include immunoregulation, metabolite production (organic acids, antimicrobial compounds, and enzymes), resident microbiota interactions, interfacing with the host, and improved gut barrier integrity (15–18). Also, the effect could be due to the regulation of blood levels of certain pro-inflammatory cytokines such as TNF-α and IL-6 (9).

Until now, several studies have already been conducted via meta-analysis of the efficacy of orally taking probiotics on the incidence reduction of diarrhea and oral mucositis in cancer patients with chemotherapy, radiotherapy, and chemoradiotherapy (14, 19, 20). However, the treatment methods for cancer patients in these studies concerned a variety of techniques, and subsequently resulted in a large heterogeneity (14, 19). Simultaneously, the data from one of the meta-analysis reports was extracted from the retrospective studies to evaluate the efficacy of probiotics on the remission of diarrhea in cancer patients with chemotherapy (20). Further, the former reports did not consider the ethnic differences that could affect the results of daily probiotic use for disease therapy. More important, several new clinical trials regarding the use of probiotics in the management of adverse reactions during chemotherapy have been published recently (9, 12, 21), which we have seriously considered in the meta-analysis. The statistical analysis demonstrated that orally administered probiotics greatly reduced the incidence of diarrhea and oral mucositis at all grades in the cancer patients with chemotherapy, but this effect was only found in Asian populations (China, India, Japan, Thailand, and Malaysia), indicating the efficacy may change in different countries because of ethnic difference. This phenomenon could be explained by the fact that the widely use of probiotics in daily healthcare, and some foods, such as cheese and yogurt, containing a great deal of probiotics are taken daily by people in Europe and America for a long history while the Asian people that begin to take probiotics as daily foods only occurs in recent decades (22). Notably, the treatment schemes including multiple species of probiotics exhibited better effects than the single bacterium in the cancer adjuvant therapy. Additionally, contrary to the former reports that the cancer patients were treated by multiple therapy methods (13, 16, 17), this meta-analysis is further confined to the adjuvant therapy of chemotherapy-induced adverse reactions in cancer patients. We fully expect that an updating meta-analysis of oral probiotic use in disease management could provide a new clinical idea for reducing the incidence of chemotherapy-induced adverse reactions.

## Materials and Methods

The systematic review and meta-analysis strictly followed the Cochrane Handbook requirements, and it had been registered in PROSPERO (CRD42020220650). We performed a preliminary search and result screening prior to registration. A systematic search and result screening has been conducted again to include all the eligible publications after registration.

### Research Question and Search Algorithm

This study was performed following the Population, Intervention, Comparator, Outcomes and Study design (PICOS)-model, which is in accordance with the Preferred Reporting Items for Systematic Reviews and Meta-Analyses (PRISMA) (23). The population was confined to the cancer patients with chemotherapy, in which the patients in the intervention group took the probiotic preparation by orally administration, and they had to be controlled (placebo or control group), but could be open-label or blinded. During the treatment, the incidences of diarrhea and oral mucositis in the cancer patients were carefully observed and recorded [Diarrhea and oral mucositis severity was graded, respectively, according to The National Cancer Institute Common Terminology Criteria for Adverse Events (CTCAE) (24) and Radiation Therapy Oncology Group (RTOG)] (25). Also, the clinical studies must be performed following the principle of randomized controlled trials. We sought to determine whether oral probiotic use could reduce the incidences of chemotherapy-induced diarrhea and oral mucositis.

### Search Strategy

We conducted a preliminary search and result screening of PubMed, Embase, ClinicalTrials.gov, and Web of Science databases on Nov. 20, 2020 to determine whether this meta-analysis could be carried out. A systematic search and result screening were performed on Dec. 31, 2021 to identify articles associated with oral probiotic use to decrease the side effects of chemotherapy in cancer patients. Literature searching was conducted by two authors (Jing Feng and Min Gao). Disagreements were resolved by discussion with a third author (Yunlei Zhang). The following keywords were used as search terms: (“Neoplasms” [MeSH Terms] OR (“Neoplasia” [Title/Abstract] OR “Neoplasms” [Title/Abstract] OR “Tumors” [Title/Abstract] OR “Tumor” [Title/Abstract] OR “Cancer” [Title/Abstract] OR “Cancers” [Title/Abstract] OR “Malignancy” [Title/Abstract] OR “Malignancies” [Title/Abstract] OR “Malignant Neoplasms” [Title/Abstract] OR “Malignant Neoplasm” [Title/Abstract] OR “Neoplasm Malignant”[Title/Abstract] OR “Neoplasms Malignant”[Title/Abstract] OR “Benign Neoplasms” [Title/Abstract] OR “Neoplasms Benign” [Title/Abstract] OR “Benign Neoplasm” [Title/Abstract] OR “Neoplasm Benign” [Title/Abstract])) AND (“Probiotics” [MeSH Terms] OR (“Probiotic” [Title/Abstract] OR “Prebiotic” [Title/Abstract] OR “Prebiotics” [Title/Abstract] OR “Symbiotic” [Title/Abstract] OR “Lactobacillus” [Title/Abstract] OR “Lactobacilli” [Title/Abstract] OR “Bifidobacterium” [Title/Abstract] OR (“Paraprobiotics” [Title/Abstract] OR “Bacterial Lysate” [Title/Abstract] OR “Postbiotics” [Title/Abstract] OR “Tyndallized” [Title/Abstract] OR “Heat-killed” [Title/Abstract])) AND (“Drug Therapy” [MeSH Terms] OR (“Therapy Drug” [Title/Abstract] OR “Drug Therapies” [Title/Abstract] OR “Therapies Drug” [Title/Abstract] OR “Chemotherapy” [Title/Abstract] OR “Chemotherapies” [Title/Abstract] OR “Pharmacotherapy” [Title/Abstract] OR “Pharmacotherapies” [Title/Abstract])) AND (“Clinical Trial” [Publication Type] OR “Intervention Study” [Title/Abstract]).

### Inclusion Criteria

The studies included in the meta-analysis were screened following the principle of PICOS: (1) Population: The studies that concern cancer patients treated by chemotherapy were considered; (2) Intervention: Cancer patients undergoing chemotherapy were given oral probiotics; (3) Comparison: Patients that did not take probiotics; (4) Outcomes: The number of patients with diarrhea or oral mucositis should be recorded; (5) Studies: The study followed the principle of randomized controlled trial.

### Exclusion Criteria

Studies were excluded if any of the following reasons were involved: (1) Studies without control groups; (2) Articles published in languages other than English; (3) Ongoing or unpublished experiments; (4) Studies with <10 patients; (5) Reviews, retrospective articles, animal experiments, independent protocols, letters, books, and personal opinions.

### Data Extraction and Risk of Bias Assessment

The data were independently extracted into pre-made tables by two of the listed authors (Chengcheng Zhao and Jian Yang). Any uncertain issues were determined by a third author (Yunlei Zhang). The data were extracted from the studies by the following terms: (1) Study characteristics: first author, publication year, country; (2) Baseline patient characteristics: patient population, cancer type, sample number, chemotherapy regimen, experimental group intervention, and comparison; (3) Outcomes measured by the number of adverse events and total number of participants; (4) The elements contributing to bias (Generation of randomization sequences, allocation concealment, participants, investigators, data assessors, integrity of outcome data, and selective outcome reporting.

### Statistical Analysis

We analyzed the data through using RevMan Statistical Software (version 5.4) and Stata Statistical Software (version 12.0). The risk ratio (RR) and 95% confidence intervals (CI) were calculated by the proportion of patients with adverse reactions in the total patients. The presence of heterogeneity was assessed through using Cochran's Q statistics and quantified by I^2^ statistics. The fixed effect model was performed if I^2^ < 50, and the random effect model was used if I^2^ > 50. The α level was set at 0.05. A *P* value of < 0.05 indicates that the differences among the groups are significant. When there was statistical heterogeneity among the studies, sensitivity analysis would be performed to identify the source of heterogeneity. The inhibitory effects of oral probiotic use on the incidence of diarrhea and oral mucositis caused by chemotherapy were further analyzed in terms of continental region, duration of intake, and number of strains.

## Results

### Literature Screening and Population Characteristics

**Figure 1:**
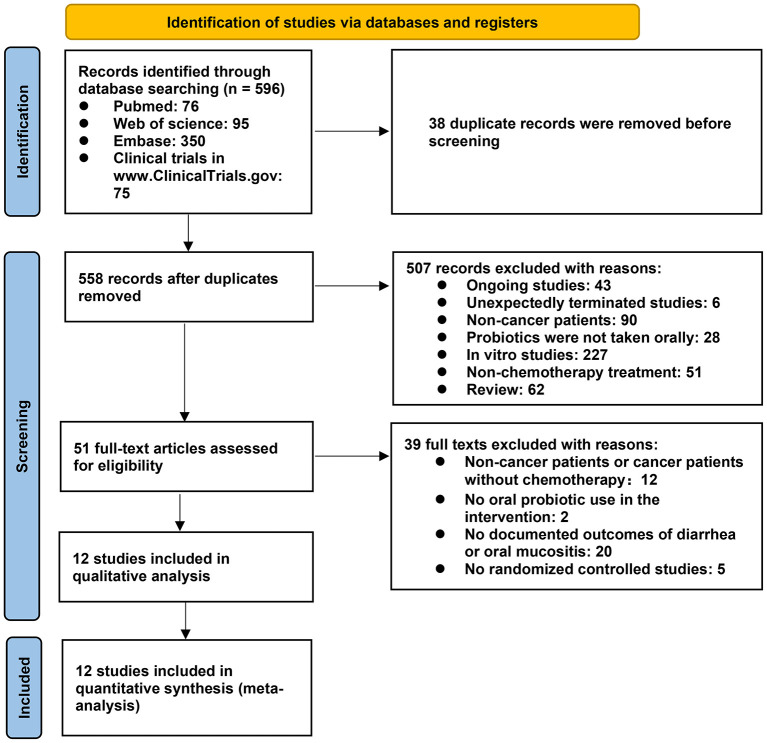
Study flow diagram.

A total of 596 articles were acquired from the databases. Following the above inclusion and exclusion criteria, 507 articles (ongoing studies, unexpectedly terminated trials, non-cancer patients, not oral probiotic use, *in vitro* studies, non-chemotherapy studies, and reviews) and 38 duplicate articles were removed after carefully reading their titles and abstract, leaving only 51 articles. After reading the remaining articles, 39 additional studies were further excluded because of that 12 studies did not include cancer patients or they were not receiving chemotherapy (26–37), two did not include oral probiotic use (38, 39), 20 had no record of diarrhea or oral mucositis in patients (40–59) (18 of them did not include diarrhea or oral mucositis indicators (40–46, 49–59), and the other two studies only recorded the frequency of diarrhea or oral mucositis rather than the number of patients who had the adverse reactions (47, 48)), and five were not randomized controlled trials (60–64). Finally, 12 articles were selected for the meta-analysis. Figure 1 describes the screening procedure, and characteristics of the 12 included studies are shown in Table 1.

**Table 1:** Characteristics of the included studies.

**References; Country**	**Age**	**Cancer type and sample number**	**Chemotherapy regimen**	**Interventions**	**Placebo-controlled**	**Outcomes**
Xia et al. (21) China	Age between 18 and 70	Locally advanced nasopharyngeal carcinoma (70)	Cisplatin	Oral probiotic cocktail containing L. plantarum MH-301, B. animalis subsp. Lactis LPL-RH, L. rhamnosus LGG-18, and L. acidophilus, or placebo	Yes	3; 4
Tian et al. (12) China	Age between 18 and 80	Lung cancer (41)	Platinum-based combination chemotherapy	Three types of *Clostridium butyricum*	Yes	1; 2
Zaharuddin et al. (9) Malaysia	Adult patient (≥ 18 years)	Colorectal cancer (14)	Combination of capecitabine and oxaliplatin	The probiotic combination (six viable microorganisms of *Lactobacillus* and *Bifidobacteria* strains)	Yes	1
Jiang et al. (65) China	The probiotic group (51.69 ± 9.79); the control group (50.40 ± 10.25)	Advanced nasopharyngeal carcinoma (93)	Cisplatin	The probiotic combination (*Bifidobacterium longum, Lactobacillus lactis*, and *Enterococcus faecium*)	Yes	3; 4
Motoori et al. (66) Sweden	The study group (62.7 ± 8.4); the control group (65.0 ± 6.7)	Advanced esophageal cancer (61)	Neoadjuvant chemotherapy consisted of docetaxel, cisplatin, and 5-fluorouracil (5-FU) (DCF therapy)	Yakult BL Seichoyaku (*Bifidobacterium breve* strain Yakult, *Lactobacillus casei* strain Shirota, and galacto-oligosaccharides) or other preparations of Biofermin	No	1; 2; 3; 4
Atul et al. (13) India	The study group (52.35 ± 9.433); the control group (52.35 ± 9.433)	Head and neck squamous cell carcinoma (188)	Cisplatin	The *Lactobacillus brevis* CD2	Yes	3; 4
Chitapanarux et al. (11) Thailand	Age between 18 and 65	Locally advanced cervical cancer (63)	Cisplatin	The probiotic combination (*lactobacillus acidophilus* and *bifidobacterium bifidum*)	Yes	2
Naito et al. (67) Japan	88 patients below the age of 70 years, and 94 patients over the age of 70 years	Superficial bladder cancer (202)	Epirubicin	Oral probiotics containing *Lactobacillus casei*	No	2
De Sanctis et al. (68) Italy	The probiotic group at the age from 34 to 74, and the control group at the age from 39 to 77	Head and neck cancer (68)	Cisplatinum and cetuximab	The *Lactobacillus brevis* CD2 lozenges	No	4
Osterlund et al. (8) Sweden	Age between 31 and 75	Colorectal cancer (148)	Mayo regimen or the simplified de Gramont regimen.	*Lactobacillus rhamnosus* GG	No	4
Mego et al. (10) Slovakia	Age between 42 and 81	Colorectal cancer (46)	Cetuximab and irinotecan	10 of lyophilized probiotic strains	Yes	1; 2
Limaye et al. (69) America	Age between 18 and 66	Locally advanced head and neck cancer (19)	TPF (docetaxel, cisplatin, and 5-fluorouracil) or PF (cisplatin, 5-fluorouracil)	Oral rinse AG013 composed of recombinant *Lactococcus lacti*	Yes	1; 4

*Outcomes: (1) Incidence of diarrhea at all grades (≥ 1 grade); (2) Incidence of severe diarrhea (≥ 2 grade); (3) Incidence of oral mucositis at all grades (≥ 1 grade); (4) Incidence of severe oral mucositis (≥ 3 grade)*.

The clinical trials used in our meta-analysis were performed in multiple countries including Italy, India, Sweden, Japan, America, Thailand, Malaysia, and China. All patients were adults (≥ 18 years old), and they were diagnosed with cancer following the clinical guidelines for cancer diagnosis in their respective countries. All the patients in the 12 clinical trials had been treated by chemotherapy. Patients in three of the studies were treated by surgery plus chemotherapy (8, 9, 67), in five studies received radiation therapy in addition to chemotherapy (11, 13, 21, 65, 68), and in the remaining studies was treated by chemotherapy alone (10, 12, 66, 69). In addition, the included patients were diagnosed with seven types of cancers, including head and neck cancer (13, 68, 69), nasopharyngeal carcinoma (21, 65), colon cancer (8–10), esophageal cancer (65), cervical cancer (11), bladder cancer (67), and lung cancer (12). The clinical trials in the 12 studies were performed following the rule of randomized controlled trials. Detailed features of these studies have been described in Table 1.

**Figure 2:**
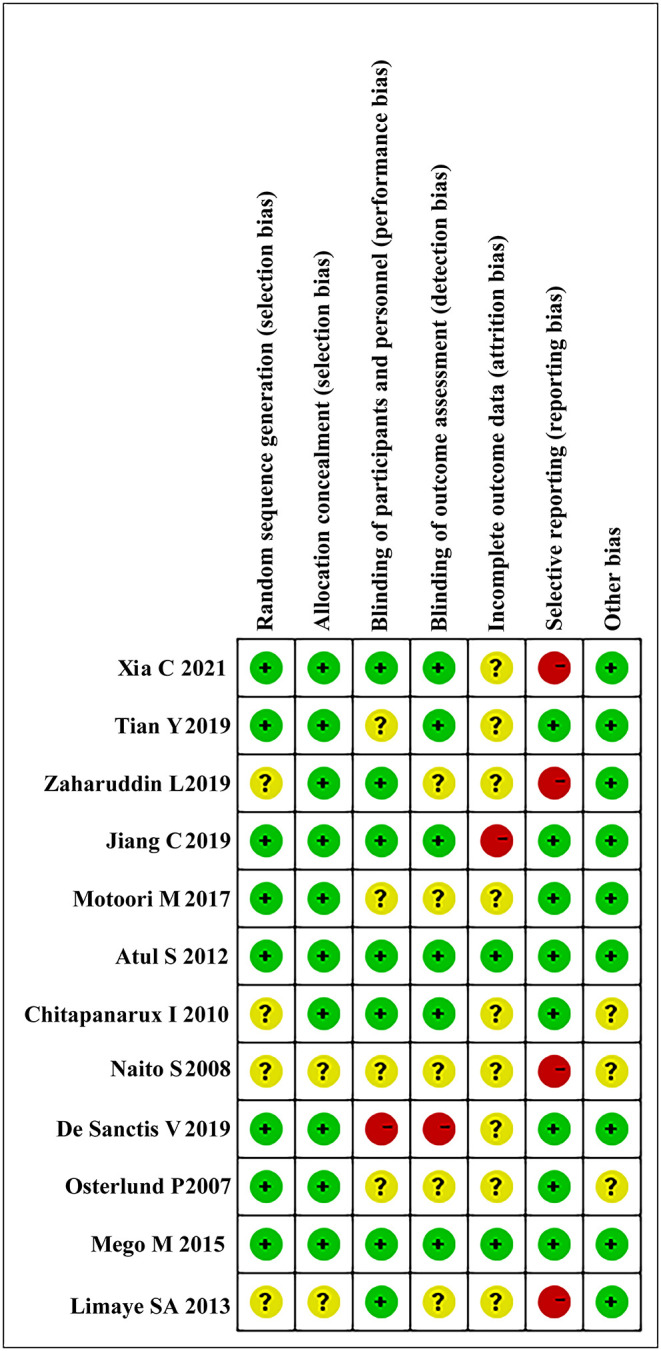
Risk of bias graph for each included study.

The risk of bias was assessed using Cochrane Collaboration's tool (70). We determined the risk of bias based on the detailed description of the treatment process in the studies. The e-mail had been sent to the authors of the studies that did not elaborate on the treatment process, and the record was labeled “unclear” in Supplementary Table 1 if there was still no enough information. The risk of bias for each article was evaluated, which has been shown in Figure 2; Supplementary Table 1.

### Oral Administration of Probiotics Improved Chemotherapy-Induced Diarrhea at All Grades

**Figure 3:**
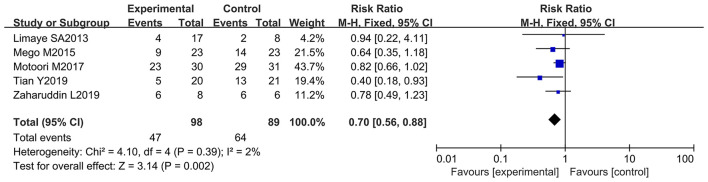
Forest plot of the efficacy of oral probiotic use against diarrhea in cancer patients.

**Figure 4:**
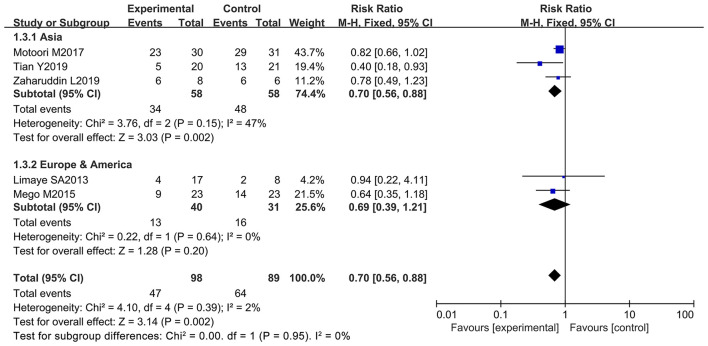
Subgroup analysis of the efficacy of oral probiotic use in the incidence reduction of diarrhea based on continental region in cancer patients.

Chemotherapy-induced diarrhea is one of the main adverse reactions induced by chemotherapy, and it occurs in most cancer patients (71). Five of the 12 articles in the meta-analysis included cancer patients with diarrhea at all grades (≥ 1 grade) (diarrhea at all grades was used as an evaluation index in the clinical trials) (9, 10, 12, 66, 69). These studies were analyzed to determine whether oral administration of probiotics could decrease side effects in cancer patients receiving chemotherapy. One report demonstrated that oral probiotic use significantly reduced the incidence of diarrhea at all grades (12), and the others did not show statistically significance (9, 10, 66, 69). The forest plot revealed that the risk of diarrhea at all grades (*RR* = 0.70; 95% Cl: 0.56, 0.88; *P* = 0.002) (Figure 3) significantly decreased because of orally taking probiotics in comparison with the control group. No significant heterogeneity was found after implementing the fixed effect model (*I*^2^ = 2%; *P* = 0.39). Then, a subgroup analysis was conducted by continental region where the clinical trials were performed. The results demonstrated that orally taking probiotics could not reduce the incidence of diarrhea at all grade in European and American populations (RR: 0.69; 95% Cl: 0.39, 1.21; *P* = 0.20). However, this side effect significantly decreased in Asian populations because of oral probiotics use (RR: 0.70; 95% Cl: 0.56, 0.88; *P* = 0.002) (Figure 4). The Egger's test (*P* = 0.301) indicated that there was no significant publication bias (Supplementary Table 2).

### Taking Probiotics Significantly Decreased the Incidence of Chemotherapy-Induced Severe Diarrhea

**Figure 5:**
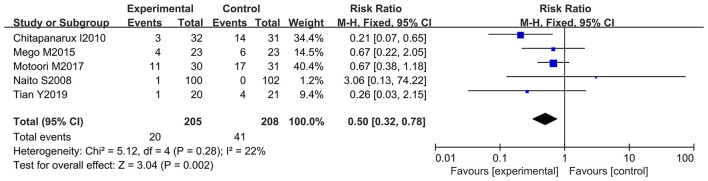
Forest plot of orally administrated probiotics for reducing severe diarrhea in cancer patients.

**Figure 6:**
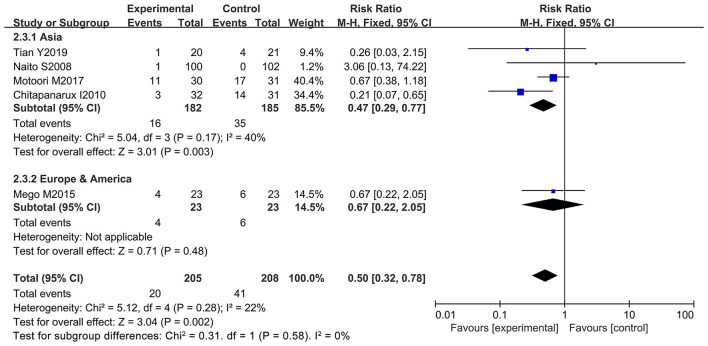
Subgroup analysis of orally administrated probiotics for reducing severe diarrhea in cancer patients based on continental region.

Further analysis was performed to observe whether oral administration of probiotics could reduce the incidence of severe diarrhea (≥ 2 grade). Patients who developed severe diarrhea during chemotherapy were recorded by five of the 12 studies (only severe diarrhea (≥ 2 grade) was used as an evaluation index in the clinical trials) (10–12, 66, 67). However, only one study clarified that oral probiotic use significantly reduced the incidence of severe diarrhea (11), and the other studies did not exhibit statistically significance (10, 12, 66, 67). Statistical analysis demonstrated that the use of probiotics decreased the severe diarrhea incidence in the cancer patients compared to the control groups (RR: 0.50; 95% Cl: 0.32, 0.78; *P* = 0.002) (Figure 5). No significant heterogeneity was found among the studies after implementing the fixed effect model (*I*^2^ = 22%; *P* = 0.28). Furthermore, the five studies were categorized and sorted into Asia (11, 12, 66, 67) and western countries (European and American) (10). Severe diarrhea incidence was clearly reduced in Asian cancer patients after oral probiotic use (RR: 0.47; 95% Cl: 0.29, 0.77; *P* = 0.003). There was no significant difference between the control groups and the experimental groups in European and America populations (RR: 0.67; 95% Cl: 0.22, 2.05; *P* = 0.48) (Figure 6). Importantly, no significant publication bias among the studies was found after conducting the Egger's test (*P* = 0.838) (Supplementary Table 3).

### Oral Administration of Probiotics Reduced Oral Mucositis Incidence at All Grades During Chemotherapy

**Figure 7:**
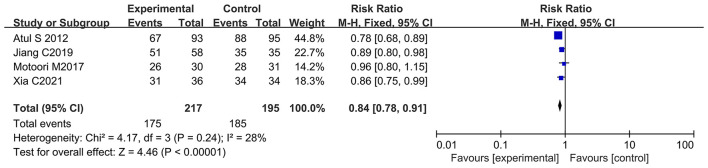
Forest plot of oral probiotic use for reducing oral mucositis in cancer patients.

Many studies reported oral mucositis as a significant adverse reaction of chemotherapy (72). Oral mucositis may lead to serious oral pain, which impairs nutritional intake, quality of life, and treatment regimens (73). Four of the 12 articles documented patients with all grades of oral mucositis (oral mucositis at all grades was used as an evaluation index in the clinical trials) due to chemotherapy (13, 21, 65, 66). Of note, three studies demonstrated that oral probiotic use significantly reduced the incidence of oral mucositis at all grades (13, 21, 65), and one did not have statistically significance (66). The forest plot indicated that oral administration of probiotics was closely associated with a lower incidence of oral mucositis in cancer patients undergoing chemotherapy (RR: 0.84; 95% Cl: 0.78, 0.91; *P* < 0.00001) (Figure 7). No significant heterogeneity was found among the studies (*I*^2^ = 28.0%; *P* = 0.24). Due to the low number of samples, the subgroup analysis by the continental region and duration of intake was not performed for the oral mucositis incidence at all grades. Besides, we did not find significant publication bias after evaluating our results with the Egger's test (*P* = 0.839) (Supplementary Table 4).

### Oral Probiotic Use Significantly Reduced Severe Oral Mucositis in Cancer Patients With Chemotherapy

**Figure 8:**
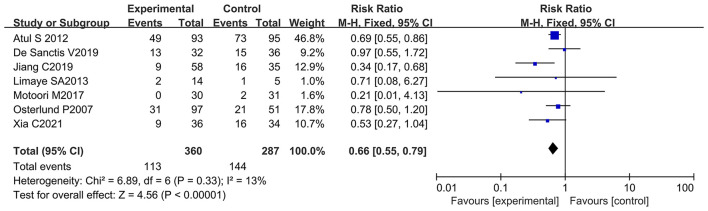
Forest plot of orally administrated probiotics for reducing severe oral mucositis in cancer patients.

**Figure 9:**
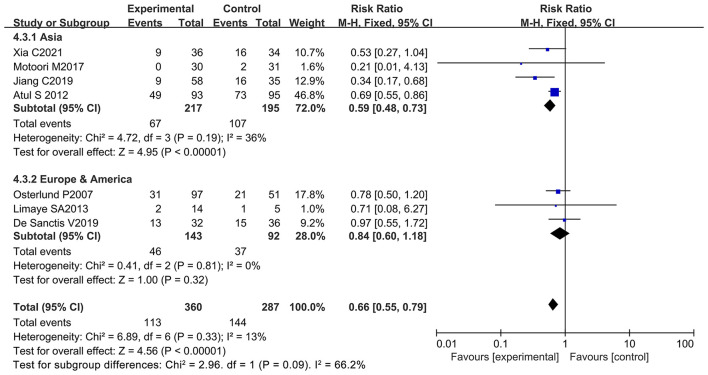
Subgroup analysis of the efficacy of oral probiotic use against severe oral mucositis in cancer patients based on continental region.

Patient data taken from seven studies were used to analyze patients with severe oral mucositis (≥ 3 grade) (only severe oral mucositis was used as an evaluation index in the clinical trials) (8, 13, 21, 66–69). Notably, two of the seven studies support the beneficial effects of oral probiotic use on the incidence reduction of severe oral mucositis (13, 65), and the other five studies did not have statistically significance (8, 21, 66, 68, 69). The forest plot signified that no significant heterogeneity occurred among the studies (*I*^2^ = 13%; *P* = 0.33). Statistical analysis showed that few patients undergoing chemotherapy developed severe oral mucositis (≥ 3 grade) because of oral probiotic use in comparison with the control group (RR: 0.66; 95% Cl: 0.55, 0.79; *P* < 0.00001) (Figure 8). The subgroup analysis further demonstrated that taking probiotics lowered the incidence of severe oral mucositis in Asian populations (RR: 0.59; 95% Cl: 0.48, 0.73; *P* < 0.00001), but not in European and American populations (RR: 0.84; 95% Cl: 0.60, 1.18; *P* = 0.32) (Figure 9). The Egger's Test (*P* = 0.450) did not find a significant publication bias among the studies (Supplementary Table 5).

### Multiple Strains Better Than the Single Bacterium in the Management of Diarrhea and Oral Mucositis in Cancer Patients With Chemotherapy

**Figure 10:**
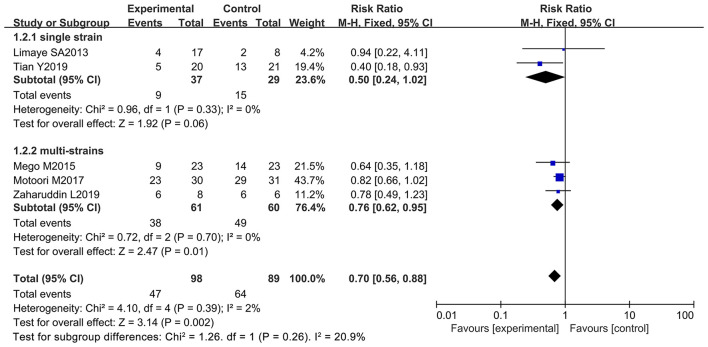
Subgroup analysis of the effect of the bacterial number on the incidence reduction of diarrhea in cancer patients.

**Figure 11:**
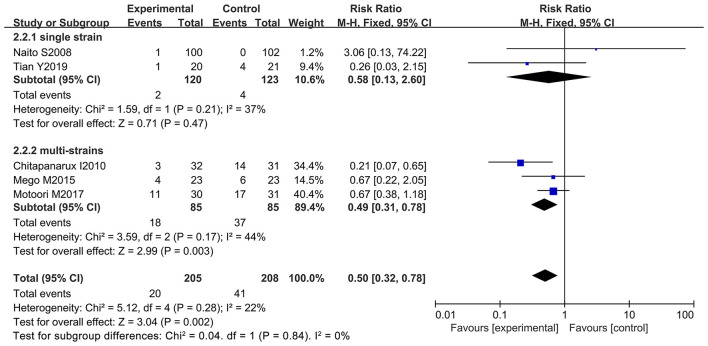
Subgroup analysis of the efficacy of bacterial number on the incidence of severe diarrhea in cancer patients.

**Figure 12:**
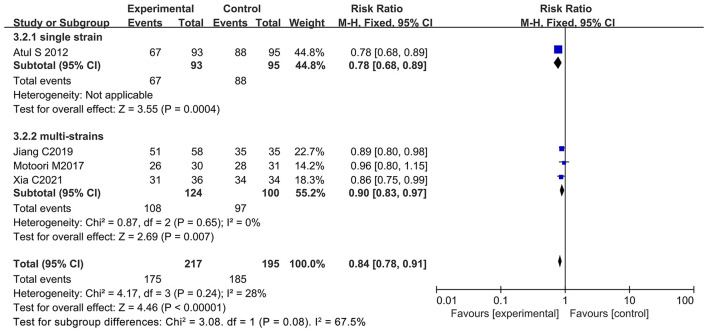
Subgroup analysis of the inhibitory effect of bacterial number on the incidence of oral mucositis in cancer patients.

**Figure 13:**
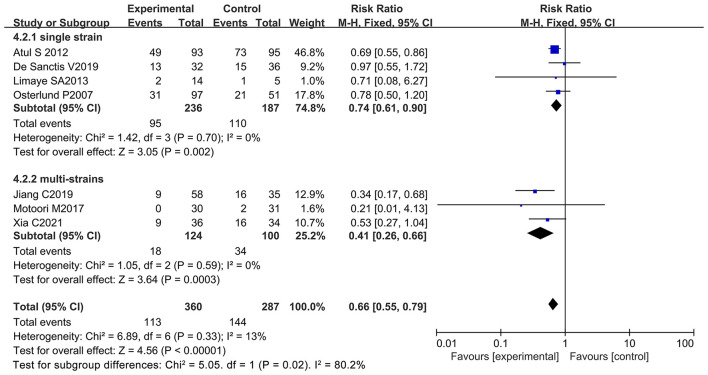
Subgroup analysis of the effect of bacterial number on the incidence reduction of severe oral mucositis in cancer patients.

Despite the beneficial effects of oral probiotic use on the reduction of diarrhea and oral mucositis during chemotherapy, the probiotics used in the 12 studies are very different in bacterial species and number. Six of the 12 studies used only one species of bacterium as active ingredient (8, 12, 13, 67–69) while other studies employed a mixture of multiple strains (9–11, 21, 65, 66) The results revealed that the treatment scheme of multiple strains significantly reduced diarrhea at all grades (RR: 0.76; 95% Cl: 0.62, 0.95; *P* = 0.01) and severe diarrhea (RR: 0.49; 95% Cl: 0.31, 0.78; *P* = 0.003). However, the single strain did not exhibit any significant effects on the incidence reduction of diarrhea at all grades (RR: 0.50; 95% Cl: 0.24, 1.02; *P* = 0.06) or severe diarrhea (RR: 0.58; 95% Cl: 0.13, 2.60; *P* = 0.47) (Figures 10, 11). Also, the treatment strategy containing multiple species of bacteria significantly decreased the incidence of oral mucositis at all grades (RR: 0.90; 95% Cl: 0.83, 0.97; *P* = 0.007) and severe oral mucositis (RR: 0.41; 95% Cl: 0.26, 0.66; *P* = 0.0003). Simultaneously, the use of single strain could also significantly lower the incidence of oral mucositis at all grades (RR: 0.78; 95% Cl: 0.68, 0.89; *P* = 0.0004) and severe oral mucositis (RR: 0.74; 95% Cl: 0.61, 0.90; *P* = 0.002) (Figures 12, 13). Collectively, oral administration of multiple strains was better than the single bacterium in the management of diarrhea and oral mucositis during chemotherapy. This could be probably due to the synergistic effects of a great number of metabolites produced by various different probiotics, the advantages of which in health management have been claimed by many studies (74, 75). The number of probiotics, strain name, drug appearance, dosage, frequency intake per day, and duration of intake are summarized in Table 2.

**Table 2:** Characteristics of the probiotics in the 12 studies.

**References; Country**	**Names of strains**	**Drug appearance and Production company**	**Dosage**	**Frequency intake per day**	**Duration of intake**
Xia et al. (21) China	*Lactobacillus plantarum* MH301, *Bifidobacterium animalis* subsp. Lactis LPL-RH, *Lactobacillus rhamnosus* LGG-18, and *Lactobacillus acidophilus*	Probiotic cocktail provided by Harbin Meihua Biotechnology Co., Ltd., Harbin, Heilongjiang, PR China	10^9^ CFU per strain	One capsule every time, and two times per day	7 weeks from the first day of chemoradiotherapy to the end
Tian et al. (12) China	*Clostridium butyricum*	Tablet produced by Qingdao East China Sea Pharmaceutical Co., Ltd, Qingdao, China	420 mg of bacteria per tablet	One tablet every time, and three times per day	About 3 weeks: the day preceding the first course of chemotherapy and the day preceding the second course
Zaharuddin et al. (9) Malaysia	*Lactobacillus acidophilus* BCMC® 12,130, *Lactobacillus lactis* BCMC® 12,451, *Lactobacillus casei* subsp BCMC® 12,313, *Bifidobacterium longum* BCMC® 02120, *Bifidbacterium bifidum* BCMC® 02290, and *Bifidobacterium infantis* BCMC® 02129	Granules provided by B-Crobes Laboratories Sdn. Bhd., Malaysia	3 × 10^11^ CFU per bacterium	Two times per day	6 months during chemotherapy
Jiang et al. (65) China	*Bifidobacterium longum, Lactobacillus lactis*, and *Enterococcus faecium*	Capsules produced by Shanghai Sine Pharmaceutical Co., Ltd, China	NA	Three capsules every time, two times a day	About 7 weeks from the beginning to the end of chemotherapy
Motoori et al. (66) Sweden	*Bifidobacterium breve* strain Yakult, and *Lactobacillus casei* strain Shirota	Yakult, and no company or institute was assigned	10^8^ CFU per bacterium	3 g of the bacteria per day	Start two days before the beginning of chemotherapy to the end of therapy
Atul et al. (13) India	*Lactobacillus brevis* CD2	Lozenges provided by CD Pharma India Pvt. Ltd	2 × 10^9^ CFU per lozenge	6 lozenges per day, one lozenge every 2–3 h	About 8 weeks from the first day of therapy to 1 week after the last treatment
Chitapanarux et al. (11) Thailand	*Lactobacillus acidophilus*, and *Bifidobacterium bifidum*	Capsule, and no company or institute was demonstrated	One capsule containing 250 mg of 10^9^ CFU *Lactobacillus acidophilus* and 10^9^ CFU *Bifidobacterium bifidum*	2 capsules per day	7 days before the treatment and continues every day during therapy
Naito et al. (67) Japan	*Lactobacillus casei* Shirota	Strain was mixed in a fermented milk, and no company or institute was indicated in the study	3 g bacteria per time	3 g per day.	Administration of the bacterial preparation was begun within 2 weeks after chemotherapy and continued for 1 year
De Sanctis et al. (68) Italy	*Lactobacillus brevis* CD2	Lozenges supplied by CD Investments Ltd, Rome, Italy	2 × 10^9^ CFU per Lozenge	6 lozenges per day, one lozenge every 2 - 3 h	About 9 weeks from the first day of treatment to the end
Osterlund et al. (8) Sweden	*Lactobacillus rhamnosus* GG	Gelatine capsules produced by Valio Ltd, Helsinki, Finland	1 - 2 × 10^10^ CFU per capsule	Twice daily.	During the 24 weeks of adjuvant cancer chemotherapy
Mego et al. (10) Slovakia	*Bifidobacterium breve* HA-129, *Bifidobacterium bifidum* HA-132 HA, *Bifidobacterium longum* HA-135, *Lactobacillus rhamnosus* HA-111, *Lactobacillus acidophilus* HA-122, *Lactobacillus casei* HA-108, *Lactobacillus plantarum* HA-119, *Streptococcus thermopilus* HA-110, *Lactobacillus brevis* HA-112, and *Bifidobacterium infantis* HA-116	Capsules produced by Harmoniom International, Inc., Mirabel, Canada	1 × 10^10^ CFU per capsule	One capsule every time, and three capsules per day	12 weeks during chemotherapy
Limaye et al. (69) America	*Lactococcus lactis* strain AG013	Liquid, and no company or institute was assigned	2 × 10^11^ CFU per 15 mL	15 mL at 1, 3, and 6 times daily	From the first 14 days of cycle 2 during the treatment

*CFU in dicates colony formation unit*.

## Conclusions

A total of twelve articles including 1,013 patients were recruited for the meta-analysis after conducting standard search and selection criteria. No significant heterogeneity or publication bias was found among the articles. Oral administration of probiotics could significantly decrease diarrhea and oral mucositis incidence in cancer patients undergoing chemotherapy compared with the control group. No death or adverse effects due to oral probiotic supplementation were recorded. In detail, seven studies reported that patients could safely ingest probiotics (8–10, 12, 13, 65, 69), and two studies demonstrated the high tolerance of patients to them (8, 69). Interestingly, six of the 12 studies used only one bacterium as active ingredient (8, 12, 13, 67–69) while other studies employed a mixture of multiple strains (9–11, 21, 65, 66) (Table 2). It should be noted that most strains used in the clinical studies, including *Lactobacillus brevis* CD2, VSL#3, and *Lactobacillus rhamnosus* GG, have already been well-studied for treating acute gastroenteritis (76), irritable bowel syndrome (77, 78), and ulcerative colitis (79). This indicates that most probiotics used to regulate intestinal microecology have potential to reduce the adverse effects in cancer therapy. Notably, the decrease of chemotherapy-induced side effects associated with probiotic use only occurred in Asian populations, not in European or American populations. This could be possibly ascribed to the ethnic difference that the people in the western countries used to daily taking probiotics that contained in the various fermented foods, resulting in the immunologic tolerance to the probiotics (22). Therefore, additional clinical studies should be conducted to thoroughly evaluate the inhibitory effects of oral probiotic use on the adverse effects caused by chemotherapy.

Moreover, it would be interesting to understand the mechanisms underlying chemotherapy-induced side effects to assist in the search of new drugs to treat cancer patients. A series of *in vitro* and *in vivo* experiments revealed that chemotherapeutic drugs induce crypt cell apoptosis as well as histopathological changes in the small intestine and colon, resulting in the alteration of intestinal absorption and subsequent diarrhea (80, 81). Chemotherapeutic drugs change the intestinal flora, destroy the dynamic balance of the intestinal tract, and lead to diarrhea (82). Also, chemotherapeutic drugs target basal epithelial cells to produce reactive oxygen species. Subsequently, mucus cells release inflammatory cytokines and induce cell apoptosis (83). Conversely, probiotics are particularly advantageous in resisting pathogenic bacteria and maintaining the intestinal microecological balance (84, 85). Specific probiotics, such as *lactobacillus*, may alter the gene expression that involved in inflammation and cell apoptosis and reverse the adverse events (86). These beneficial effects of probiotics possibly contribute to the incidence reduction of adverse reactions caused by chemotherapy in some cancer patients of the included studies.

However, small samples (1,013 patients) in the meta-analysis are not sufficient to fully assess the beneficial effects of oral probiotic use on the incidence reduction of adverse reactions in cancer patients during chemotherapy. Additionally, not all patients in the meta-analysis strictly adhered to a single treatment by chemotherapy. Some patients were treated with two clinical methods, such as chemoradiotherapy or surgery plus chemotherapy. Simultaneously, eight of the included 12 studies used placebo as the control (9–13, 21, 65, 69), and the remaining studies just used other bacteria or non-treatment as the control (8, 66–68). More important, despite the statistical results of data extracted from the 12 studies revealed the inhibitory effects of oral probiotic use on the incidence of diarrhea and oral mucositis during chemotherapy, only two studies demonstrated the relieve of diarrhea while three studies indicated the incidence reduction of oral mucositis in the cancer patients. Simultaneously, the species and number of strains used in the clinical trials of the meta-analysis were totally different, indicating the imperative requirement to further explore the valuable probiotics and the preparation of multi-strain combination in the management of these chemotherapy-induced side effects. Of note, considering the negative result caused by ethnic difference in the meta-analysis, probiotic species should be also seriously considered when use them to assist cancer treatment in different ethnicities. Thus, more clinical trials and basic researches are needed to fully address these limitations prior to clinical application of the probiotics.

Collectively, this meta-analysis presented the efficacy of orally administered probiotics in the incidence reduction of diarrhea and oral mucositis in cancer patients during chemotherapy based on multiple clinical trials, and the mechanism underlying the action was also discussed. Considering the crucial role of chemotherapy in cancer treatment, the conclusion of the meta-analysis could provide some suggestions for people to control chemotherapy-induced adverse effects. Future studies should further evaluate the efficacy of probiotics in the management of adverse reactions in cancer patients during chemotherapy through conducting more clinical trials, and also determine the most effective probiotic species, the doses of each strain, and the dosing schedule for probiotic use in alleviating the side effects.

## Data Availability Statement

The original contributions presented in the study are included in the article/Supplementary Material, further inquiries can be directed to the corresponding authors.

## Author Contributions

YZ, JF, and MG conceived and designed the research. YZ had primary responsibility for final content, established eligibility criteria and search strategy. JF and MG conducted the database search and screened. JY and CZ worked on literature selection, data extraction and quality assessment. JF, HG, XL, and XZ performed statistical analysis. MG, RJ, and CZ prepared the figures and tables. All authors read the manuscript and approved the final draft.

## Funding

This research was supported by the National Natural Science Foundation of China (grant number: 81971726).

## Conflict of Interest

The authors declare that the research was conducted in the absence of any commercial or financial relationships that could be construed as a potential conflict of interest.

## Publisher's Note

All claims expressed in this article are solely those of the authors and do not necessarily represent those of their affiliated organizations, or those of the publisher, the editors and the reviewers. Any product that may be evaluated in this article, or claim that may be made by its manufacturer, is not guaranteed or endorsed by the publisher.
