# Supplementary material for: Oral Administration of Probiotics Reduces Chemotherapy-Induced Diarrhea and Oral Mucositis: A Systematic Review and Meta-Analysis
Source: Front Nutr. 2022 Feb 28;9:823288. doi: 10.3389/fnut.2022.823288 (PMC8922230; doi:10.3389/fnut.2022.823288)
Supplement: Supplementary file 1 [file Data_Sheet_1.zip › Data Sheet 1.PDF]

Supplementary Materials

Supplementary Table S1. Risk bias of included studies

Author; year; country	Generation of randomization sequence	Allocation concealment	Participants	Investigators	Data assessors	Integrity of outcome data	Selective outcome reporting
[21]Xia C. 2021 China	Low Risk: The random allocation sequence was performed at a ratio of 1:1 using a pseudo-random number generator.	Low Risk: The randomization sequence was produced before the first enrollment.	Low Risk: About 77 patients were finally selected and randomized (1:1) to receive either a probiotic mixture or a placebo.	Low Risk: Patients with nasopharyngeal carcinoma were assigned to the probiotic cocktail group or the control group by the clinical research technician who was also blinded.	Unclear Risk：It is not mentioned in the article.	High Risk: probiotics group (n = 39) lost 3; placebo group (n = 38) lost 4.	Low Risk: There was no selective reporting according to the clinical registration information posted (Clinical Trials number NCT03112837).
[12]Tian Y. 2019 China	Low Risk: The patients were randomly placed at the study group or the placebo group using a random number table.	Low Risk: The patients were randomly placed at the study group or the placebo group using a random number table.	Unclear Risk：It is not mentioned in the article.	Low Risk: The treatments were administered by assistants who were not aware of whether the drugs were placebo or not.	Unclear Risk：It is not mentioned in the article.	Low Risk: 50 patients were enrolled, and 5 patients were ineligible for analysis.	Low Risk: There was no selective reporting according to the clinical registration information posted (Clinical Trials number

							NCT02771470)
[9]Zaharuddin L. 2019 Malaysia	Unclear Risk：It is not mentioned in the article	Low Risk: Recruited patients were randomized through simple randomization into either treated with probiotic or placebo. Unblinding was done upon completion of data analysis.	Low Risk: Placebo samples produced were identical to the probiotics in terms of taste and texture except it did not contain any live microorganisms.	Unclear Risk：It is not mentioned in the article.	Unclear Risk：It is not mentioned in the article.	High Risk: 75 patients were enrolled, and 23 patients were ineligible for analysis.	Low Risk: There was no selective reporting according to the clinical registration information posted (Clinical Trials number NCT03782428).
[66]Jiang C. 2019 China	Low Risk: The random assignment of patients was performed through a computer-generated random number code.	Low Risk: The blinding codes for the seeds of the random numbers, the block length, and the random numbers, were sealed in envelopes and stored at Jiangxi Cancer Hospital. Blinding codes were not disclosed during the entire trial period.	Low Risk: The shape and color of the placebo as well as other properties were identical to the probiotic combination.	Low Risk: Patients were randomly distributed into 6 blocks in a 2:1 ratio to receive probiotics or a placebo (the block size was known only to the statistician).	High Risk: The block size was known only to the statistician.	Low Risk: Study group (n = 64) lost 6; Control group (n = 35) lost 0	Low Risk: There was no selective reporting according to the clinical registration information posted (Clinical Trials number NCT03112837).

[69]Motoori M. 2017 Sweden	Low Risk: Data center in the Department of Surgery Osaka Medical Center for Cancer and Cardiovascular Diseases generated a randomization table.	Low Risk: Randomization was performed by permuted block method with block sizes of 4.	Unclear Risk: It is not mentioned in the article.	Unclear Risk: It is not mentioned in the article.	Unclear Risk: It is not mentioned in the article.	Low Risk: All 61 patients were included in the intention-to-treat analysis.	Low Risk: There was no selective reporting according to the University Hospital Medical Information Network (number UMIN000006875) .
[13]Atul S. 2012 India	Low Risk: Patients were randomly assigned to either of the treatment arms in a 1:1 ratio through a computer-generated randomization list consisting of randomly permuted blocks of 10 patient numbers.	Low Risk: Patients were randomly assigned to either of the treatment arms in a 1:1 ratio through a computer-generated randomization list consisting of randomly permuted blocks of 10 patient numbers.	Low Risk: The *Lactobacillus brevis* lozenges and placebo were supplied by CD Pharma India Pvt. Ltd. and were identical in physical appearance, taste and color.	Low Risk: The study products were pre-packaged by the sponsor as per the randomization codes and dispensed accordingly.	Low Risk: An independent biostatistician analyzed the study data.	Low Risk: Study group (n = 101) lost 8; Control group (n = 99) lost 4	Low Risk: There was no selective reporting according to the clinical registration information posted (Clinical Trials number CTRI/2008/ 091/000117).
[11]Chitapanarux I.	Unclear Risk: It is not mentioned in the article.	Low Risk: Patients were randomly	Low Risk: The placebo had the same size and	Low Risk: Pre-packaged (blinded) study medication	Unclear Risk: It is not mentioned in	Low Risk: All 63 patients were eligible and assessable.	Unclear Risk: It is not mentioned in the article.

2010 Thailand		assigned in a double blind fashion to receive study drug or placebo in a 1: 1 ratio.	color as the study drug.	differing solely in the patient numbers on the medication package was provided by the sponser.	the article.		
[65]Naito S. 2008 Japan	Unclear Risk: It is not mentioned in the article	Unclear Risk: It is not mentioned in the article.	Unclear Risk：It is not mentioned in the article.	Unclear Risk：It is not mentioned in the article.	Unclear Risk：It is not mentioned in the article.	High Risk: 207 patients were enrolled, and 5 patients were ineligible for analysis.	Unclear Risk：It is not mentioned in the article.
[67]De Sanctis V. 2019 Italy	Low Risk: Patients were randomised to standard oral care regimen with control arm or intervention arm in a 1:1 ratio through a computer-generated randomization list consisting of randomly permuted blocks of four patient	Low Risk: Patients were randomised to standard oral care regimen with control arm or intervention arm in a 1:1 ratio through a computer-generated randomization list consisting of randomly permuted blocks of four patient	High Risk: The appearance and taste of *Lactobacillus brevis* CD2 lozenges was different from sodium bicarbonate mouthwash.	High Risk: The appearance and taste of *Lactobacillus brevis* CD2 lozenges was different from sodium bicarbonate mouthwash.	Unclear Risk: It is not mentioned in the article.	Low Risk: 75 patients were enrolled, and 7 patients were ineligible for analysis.	Low Risk: There was no selective reporting according to the clinical registration information posted (Clinical Trials number NCT01707641).

	numbers.	numbers.					
[8]Osterlund P. 2007 Sweden	Low Risk: Allocation to the study treatments was performed using a computerised minimisation technique and one out of six chances.	Low Risk: The allocation group was concealed until interventions had been assigned.	Unclear Risk: It is not mentioned in the article.	Unclear Risk: It is not mentioned in the article.	Unclear Risk：It is not mentioned in the article.	Low Risk: 154 patients were enrolled, and 4 patients were ineligible for analysis.	Unclear Risk: It is not mentioned in the article.
[10]Mego M. 2015 Slovakia	Low Risk: Patients were allocated to one of the treatment group (probiotic or placebo) based on preformed randomization table.	Low Risk: Patients were centrally randomized in a ratio 1:1. Patients were allocated to one of the treatment group (probiotic or placebo) based on preformed randomization table.	Low Risk: All containers with probiotics/placebo looked the same and were sequentially numbered. The placebo was indistinguishable from the capsule with probiotics in terms of color, appearance, taste, smell, shape and other properties and contained the	Low Risk: Investigator received the identification number of containers for randomized patient and patient received corresponding containers.	Low Risk: Patients, investigators and statisticians were blinded to treatment allocation.	Low Risk: 49 patients were enrolled, and 3 patients were ineligible for analysis.	Low Risk: There was no selective reporting according to the clinical registration information posted (Clinical Trials number NCT01410955)

			same additives as probiotic capsule.				
[68]Limaye SA. 2013 America	Unclear Risk: It is not mentioned in the article.	Unclear Risk: It is not mentioned in the article.	Low Risk: The study subjects were unaware of the study arm they were on.	Unclear Risk: It is not mentioned in the article.	Unclear Risk: It is not mentioned in the article.	High Risk: Study group (n = 17) lost 3; Control group (n = 8) lost 3.	Low Risk: There was no selective reporting according to the clinical registration information posted (Clinical Trials number NCT00938080).

Supplementary Table S2. Published bias of oral probiotics in diarrhea studies

**Std_Eff**	**Coef.**	**Std. Err.**	**t**	**P > |t|**	**[95% Conf. Interval]**	
Slope	-.1173117	.1375672	-0.85	0.456	-.5551119	.3204885
Bias	-.8302639	.6662068	-1.25	0.301	-2.950431	1.289903

Supplementary Table S3. Published bias of oral probiotics in severe diarrhea studies

Egger's test	Coef.	Std. Err.	t	P > |t|	95% Cl	
Slope	-.4833241	.5792014	-0.83	0.465	-2.326602	1.359953
Bias	-.252233	1.131851	-0.22	0.838	-3.854288	3.349822

Supplementary Table S4. Published bias of oral probiotics in oral mucositis studies

**Std_Eff**	**Coef.**	**Std. Err.**	**t**	**P > |t|**	**[95% Conf. Interval]**	
Slope	-.2005771	.2379562	-0.84	0.488	-1.22442	.8232659
Bias	.8063838	3.490359	0.23	0.839	-14.21142	15.82419

Supplementary Table S5. Published bias of oral probiotics in severe oral mucositis studies

**Std_Eff**	**Coef.**	**Std. Err.**	**t**	**P > |t|**	**[95% Conf. Interval]**	
Slope	-.2818262	.1632714	-1.73	0.145	-.7015287	.1378763
Bias	-.5704603	.6966863	-0.82	0.450	-2.361349	1.220429
